# Anemia on Admission Is an Independent Predictor of Long-Term Mortality in Hip Fracture Population: A Prospective Study With 2-Year Follow-Up

**DOI:** 10.1097/MD.0000000000002469

**Published:** 2016-02-08

**Authors:** Licheng Zhang, Pengbin Yin, Houchen Lv, Anhua Long, Yuan Gao, Lihai Zhang, Peifu Tang

**Affiliations:** From the Department of Orthopedics, General Hospital of Chinese PLA, Beijing (LZ, PY, HL, AL, YG, LZ, PT); and School of Medicine, Nankai University, Tianjin, China (AL).

## Abstract

Anemia is a disputable factor for long-term mortality in hip fracture population in previous studies. Previous studies indicated that the level of hemoglobin (Hb) might fluctuate due to various factors, such as comorbidities and in-hospital interventions, and the changing level of Hb, may lead to discordance diagnosis of anemia and thus to the conflicting conclusions on prognostic value of anemia. So in this study, we aim to compare factors affecting the diagnosis of anemia at different time-points, admission, postoperation, and discharge, and to determine which the time point is most suitable for mortality prediction.

This prospective cohort study included 1330 hip fracture patients from 1 January 2000 to 18 November 2012. Hb levels at 3 different time points, such as admission, postoperation, and discharge, were collected and used to stratify the cohort into anemia and nonanemia groups. Candidate factors including commodities, perioperative factors, blood transfusion, and other in-hospital interventions were collected before discharge. Logistic regression analyses were performed to detect risk factors for anemia for the 3 time points separately. Kaplan–Meier and multivariate Cox regression analyses were used to evaluate the association between anemia and 2-year mortality.

Factors affecting the diagnosis of anemia were different for the 3 time points. Age, female sex, American Society of Anesthesiologists score (ASA), and intertrochanteric fracture were associated with admission anemia, while surgical procedure, surgical duration, blood transfusion, blood loss during the operation, and drainage volume were major risk factors for postoperation anemia. Cox proportional-hazards regression analysis suggested that the risk of all-cause mortality was higher in the anemia group on admission (1.680, 95%CI: 1.201–2.350, *P* < 0.01), but not postoperation or on discharge, after adjustment for confounding factors.

Our study showed that risk factors for anemia varied at different time points, and therapy interventions would greatly affect the status of postoperation and discharge anemia in hip fracture patients. The take-home message is when anemia is used for mortality prediction in these patients, a specific time point should be chosen. We suggest that only admission anemia should be used for mortality prediction, but not postoperation nor discharge anemia.

## INTRODUCTION

Hip fracture has become an increasingly severe disease with reported mortality rates ranging from 8.4% to 36%.^1–3^ Prior studies pointed out various risk factors, such as age, sex, fracture type, blood transfusion, and surgical procedure, which may be associated with mortality and outcome of this fracture.^4–9^ These factors help identify populations at a higher risk of death or poor outcomes, and therefore may benefit patients by increasing the vigilance of doctors when making clinical decisions.

Recently, several studies reported close associations between anemia and short- and long-term mortality. Vochteloo et al^10^ showed that a population with low hemoglobin (Hb) levels exhibited a higher risk of 3- and 12-month mortality. Bhaskar and Parker^11^ also demonstrated that patients with Hb levels ranging from 80 to 100 g/L (normal range is ≥130 g/L for men and ≥120 g/L for women) were at higher risk of death. However, conflicting results were reported by Mahdian and coworkers,^4^ who found no association between low Hb and mortality. Similarly, Hagino et al^12^ identified anemia as related to age, fracture type, and walking ability, but not mortality. Thus, whether anemia is indeed associated with hip fracture mortality has remained controversial, and the causes behind the inconsistencies of previous studies must be addressed.

Prior research has shown that Hb levels fluctuate during a surgical patient's hospital course. Halm et al^13^ reported that during the entire hospitalization, the average decline in Hb was 15 ± 18 g/L. Similarly, Wang et al^14^ confirmed that Hb and hematocrit dropped in hip fracture patients after intramedullary nailing. Risk factors contributing to this phenomenon remain undetermined. Nonetheless, the results of these studies suggest that the timing of anemia diagnosis may be critical to its application for mortality prediction. Therefore, we designed this prospective study to detect whether the timing of anemia diagnosis affected its application for the risk prediction of hip fracture mortality.

The objective of our study was first to compare factors affecting the diagnosis of anemia at different time-points, admission, postoperation, and discharge, and then to validate which time point is most suitable for predicting long-term all-cause mortality in hip fracture patients. This study aimed to address previous conflicting results of the association of anemia and long-term outcome, and might do help to further use of anemia as a prognostic variable in hip fracture patients.

## METHOD

### Study Design and Settings

**FIGURE 1:**
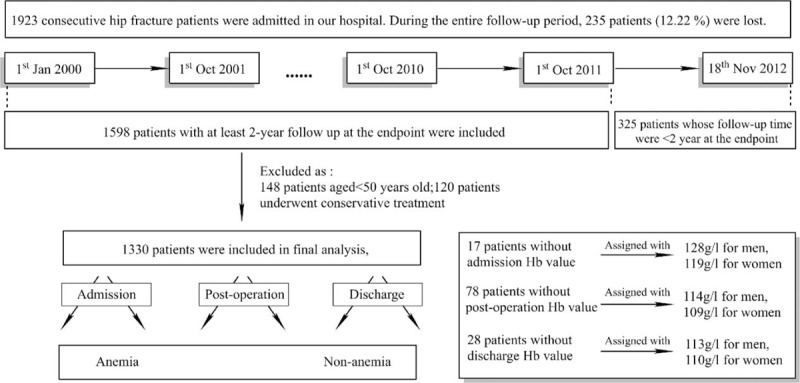
Flowchart indicating patient inclusion.

This study is a part of Chinese PLA General Hospital Hip Fracture Study (PLAGH Hip Fracture Study), a single-center, prospective cohort study which was undertaken at the Chinese PLA General Hospital. The PLAGH Hip Fracture Study included 1923 consecutive hip fracture patients admitted to our hospital from 1 January 2000 to 18 November 2012. During the entire follow-up period, 235 patients (12.22% of 1923) were lost. In order to ensure that all patients in this study had at least a 2-year follow-up at the study endpoint (1 December 2013), 1598 patients between 1 January 2000 and 1 October 2011 were extracted for further analysis. Diagnosis of hip fracture, including femoral neck fracture and intertrochanteric fracture (International Classification of Diseases [ICD] codes [820 for the 9th version]), was based on radiographic examination including X-ray and/or computer tomography. Patients with pathological fractures, aged <50 years, who underwent conservative treatment (no surgery), or who were lost to follow-up before 2 years were excluded from the study (Figure 1). For patients with bilateral hip fractures, data from their 1st admission were collected. Ultimately, 1330 patients were included in the final cohort for analysis.

All-cause mortality data were collected 2 years after the discharge date by reviewing the hospital registry and medical notes, or by telephone survey. This study was performed in compliance with the World Medical Association Declaration of Helsinki and was approved by the medical ethics committee of PLA General Hospital.

### Variables

Sample size calculation: we used data from 2000 to 2008 to calculate the incidence of anemia (approximately 50%) and 2-year all-cause mortality of anemic (approximately 20%) and nonanemic (approximately 10%) hip fracture patients for further calculation of sample size. Alpha (the probability of rejecting a true null hypothesis) was set as 0.05, power (the probability of rejecting a false null hypothesis) was 0.9, and the proportion of patients lost during follow-up was set as 10%. A 2-side log rank test indicated that at least 606 subjects should be included into the cohort. In this study, we included 1330 patients for further analysis.

Fracture classification (femoral neck fracture and intertrochanteric fracture), surgery type (simple fixation or external fixation, intramedullary, arthroplasty, and others [extramedullary, plate fixation], blood transfusion, surgery duration, blood loss during the operation, and drainage volume were collected before discharge. The complete blood cell count results (XE-2100, Sysmex Corporation, Kobe, Japan) on admission, postoperation, and on discharge were all recorded. Among the study cohort, 17 patients’ Hb levels on admission, 78 patients’ Hb levels postoperation, and 28 patients’ Hb levels on discharge were missing. Because the Hb value was a normally distributed variable, a mean Hb value for each time point was assigned to these patients according to their sex (128 g/L for men, 119 g/L for women on admission; 114 g/L for men, 109 g/L for women on postoperation; and 113 g/L for men, 110 g/L for women on discharge).

A Charlson comorbidity index (CCI) score was assigned to each patient on admission to describe comorbidity burdens. The international classification of diseases coding algorithms, version 9 (ICD-9) developed by Deyo (2005), was used to generate the CCI, because it appears to be the most widely used in the literature.

### Statistical Analysis

Statistical description of the entire cohort was performed before analysis. Normally distributed variables are presented as mean ± standard deviation, while median with quartile is shown for nonnormally distributed variables. For categorical variables, the number of patients in each category was counted and recorded.

Univariate logistic regression analyses were first performed to detect the risk factors (clinically relevant variables including sex, age, CCI score, and classification of fracture, surgical procedures, and anesthesia) for anemia at different time points. These factors were then included in multivariate logistic regression analyses (backward) to detect factors affecting the diagnosis of anemia. The survival curve for patient mortality was constructed using Kaplan–Meier analysis, and the log-rank test was used to test for differences within subgroups. Cox proportional hazards models were used to assess the prognostic role of anemia, demonstrated by calculating hazard ratios and corresponding 95% confidence intervals (95% CIs), and the proportional hazards assumption was tested by Walds test. All statistical analyses were performed using the Statistical Product and Service Solutions (SPSS) 19.0 software (IBM Corporation, Armonk, NY). *P* values <0.05 were considered significant.

## RESULTS

### Baseline Characteristics of the Study Population

This study was based on the database of PLAGH Hip Fracture Study from 1 January 2000 to 18 November 2012. Of 1598 patients with at least 2-year follow-up, those aged <50 (n = 148) or underwent conservative treatment (n = 120) were excluded from the study cohort. At last, 1330 patients were included for analysis (Figure 1).

The baseline demographic characteristics of the cohort are shown in Table 1. The median age was 76 years (interquartile range, 69, 82), with 504 men and 826 women. Ninety three patients were graded 1 point based on the CCI, meanwhile 176 patients with 2 points, 649 patients with 3 points, 232 patients with 4 points, and the remainder (n = 180) with 5 or more points. Blood transfusion was performed in 995 patients during the entire hospitalization period, 335 patients did not receive a transfusion. Intertrochanteric fracture was diagnosed in 722 patients, and the rest (n = 608) was diagnosed with femoral neck fracture. A total of 484 patients received intramedullary fixation, 652 underwent hip arthroplasty, and the remainder (n = 194) received other surgical interventions. A total of 984 patients were injured by high-impact trauma such as a car accident or falling from a height, and 346 were injured by low-impact trauma such as a sprain or tripping from a standing position. The mean Hb level on admission was 121.0 ± 20.8 g/L; postoperation, 110.5 ± 16.7 g/L; and on discharge, 111.3 ± 19.1 g/L. According to WHO criteria for the diagnosis of anemia, patients were divided into anemia and nonanemia groups at each time point as shown in Table 1.

**TABLE 1:**
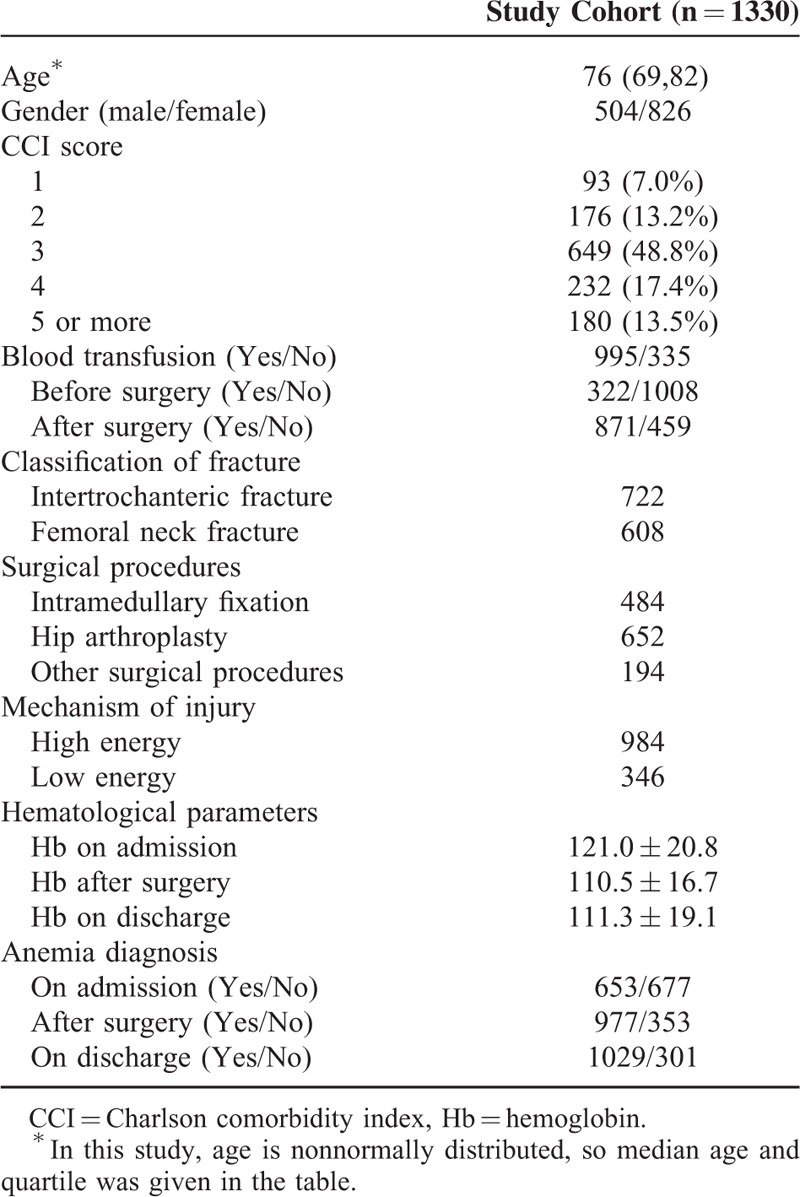
Baseline Demographic Characteristics

### Main Outcomes

Anemia was present in 49.1% of patients on admission. On postoperation, 73.5% of patients demonstrated anemia, among whom 386 patients had not been diagnosed with anemia on admission. In addition, 77.4% patients were anemic on discharge, among whom 443 of these patients did not present anemia on admission. Sixty-two and 67 patients, respectively, who presented anemia on admission became nonanemic postoperation and on discharge.

## FACTORS ASSOCIATED WITH ANEMIA AT DIFFERENT TIME POINTS

### Univariate Analyses

Univariate logistic regression analyses were performed to detect factors that may be associated with anemia at different time points. Age >80 years, male sex, CCI score, and intertrochanteric fracture were identified as risk factors for anemia on admission, with details of OR ratio described in Table 2. On postoperation, associations were found between inpatient interventions (surgical procedure, surgical duration, blood transfusion, and blood loss during operation) and anemia (all *P* < 0.01). However, except for fracture classification, no prehospital variables were associated with postoperative anemia. Surgical duration, blood transfusion, and drainage volume did not show any significant relationship with anemia on discharge, while other inpatient interventions remained as risk factors for anemia. Male sex and intertrochanteric fracture were also associated with anemia on discharge.

**TABLE 2:**
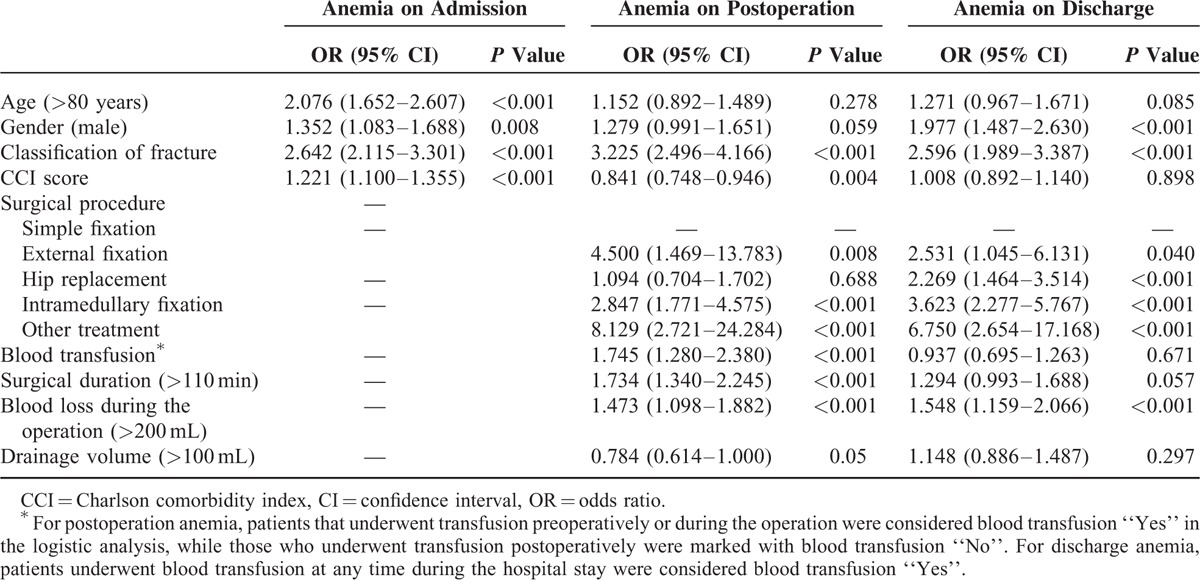
Univariate Logistic Regression of Risk Factors at Different Time Points

### Multivariate Analyses

Variables were then included in multivariate logistic regression analyses (backward). Age >80 years, male sex, CCI score, and intertrochanteric fracture remained as risk factors for admission anemia (all *P *< 0.01). Meanwhile blood transfusion, surgical duration over 110 min, and blood loss during operation >200 mL, as well as intertrochanteric fracture, showed significant associations with postoperation anemia in the multivariate model (all *P* < 0.05). For discharge anemia, male sex, intertrochanteric fracture, and blood loss >200 mL were shown as risk factors (all *P* < 0.05).

## ASSOCIATION OF ANEMIA AND 2-YEAR ALL-CAUSE MORTALITY

### Survival Curves of the Anemia and Nonanemia Groups at Three Time Points

**FIGURE 2:**
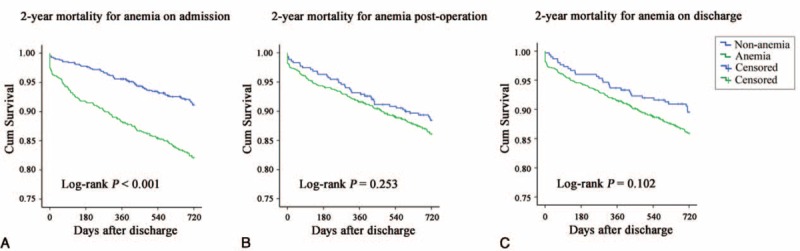
The survival curve for 2-year mortality of patients with hip fracture. (A) 2-year mortality for anemia on admission. All-cause 2-year mortality in anemic patients was significantly higher than nonanemic patients by Log-rank test. (B) 2-year mortality for anemia on postoperation. No significant difference was found between anemic and nonanemic patients. (C) 2-year mortality for anemia on discharge. No significant difference was found between anemic and nonanemic patients.

Kaplan–Meier analysis revealed a significant increase in the probability of death for patients in the anemia group on admission (log-rank *P* < 0.001), as shown in Figure 2. The 2-year mortality rates were 18.1% and 8.6% for the anemia and nonanemia groups on admission, respectively. However, no difference was found between the anemia and nonanemia groups postoperation or on discharge (postoperation, log-rank *P* = 0.253; discharge, *P* = 0.102).

### Anemia on Admission Is an Independent Risk Factor for Hip Fracture Mortality

**FIGURE 3:**
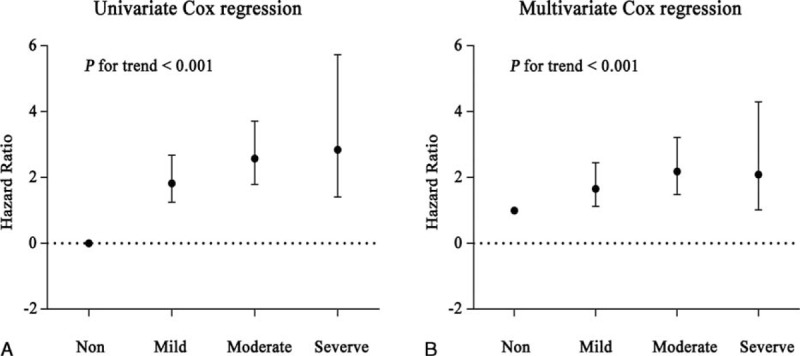
Hazard ratio of varying degree of on admission anemia. (A) Hazard ratio of varying degree of on admission anemia calculated by univariate Cox proportional regression, severe anemia had higher HR than nonanemia (*P* for trend <0.001). (B) Hazard ratio of varying degree of on admission anemia calculated by multivariate Cox proportional regression, adjusting variables in Table 4. Severe anemia had higher HR than nonanemia (*P* for trend <0.001).

As shown in Table 4 and Figure 3, Cox proportional-hazards regression analysis suggested that the risk of mortality was higher in the anemia group on admission, compared with the nonanemia group (*P* < 0.001). This difference persisted even after adjustment for sex, age, CCI score, and classification of fracture, surgical procedures, and anesthesia (*P* < 0.001). Further classification of anemia based on Hb level on admission was then applied to the cohort based on WHO criteria for mild anemia (men 110–129 g/L, women 110–119 g/L), moderate anemia (men and women 80–109 g/L), and severe anemia (men and women <80 g/L). Mortality odds ratios compared to the reference category (nonanemia group) are shown in Table 3. Patients with mild anemia on admission exhibited a 1.8-fold increase in mortality risk (95% CI: 1.250–2.675), those with moderate anemia a 2.5-fold increase (95% CI: 1.792–3.712), and those with severe anemia a 2.8-fold increase (95% CI: 1.407–5.731) (all *P* < 0.01). Further adjustment with prior risk factors only partially attenuated the effect of anemia with 2-year all-cause mortality (Table 4).

**TABLE 3:**
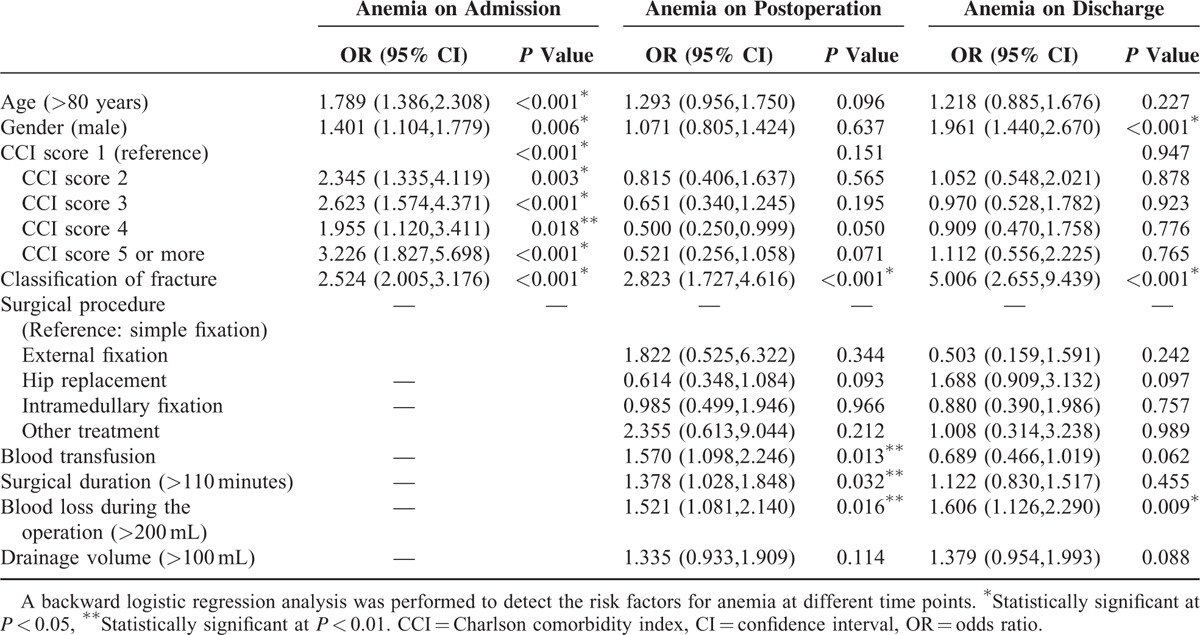
Multivariate Logistic Regression of Risk Factors for Anemia at Different Time Points

**TABLE 4:**
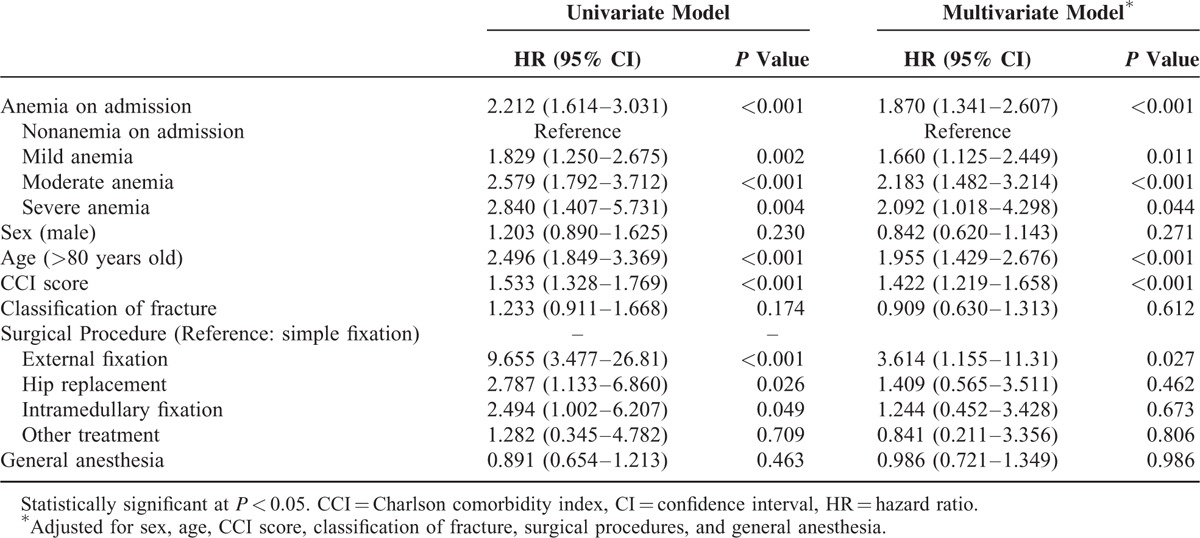
Hazard Ratios for 2-year Mortality According to Anemia on Admission

## DISCUSSION

The primary finding of our study was that risk factors for inpatient anemia vary with the time point of diagnosis. Prehospital variables were significantly associated with admission anemia, while inpatient interventions were the major risk factors for postoperation and discharge anemia. Second, anemia on admission, not postoperation or discharge, was an independent risk factor for all-cause mortality in these patients. These results could address previous conflicting results of the association of anemia and long-term outcome, and do help to further use of anemia as a prognostic variable in these patients.

According to the WHO, anemia is a condition in which the number of red blood cells or their oxygen-carrying capacity is insufficient to meet physiological needs, which varies by age, sex, altitude, smoking, and pregnancy status.^15^ Normally, anemia is the result of a wide variety of coexistent causes, such as nutrition deficiency and chronic diseases. Beyond that, trauma-related factors may affect hemodynamics, also causing acute anemia.^16^ Our study showed that age, female sex, CCI score, and intertrochanteric fracture were associated with anemia on admission. Prior research by Patel^17^ indicated that after the age of 50 years, the prevalence of anemia increases with advancing age and exceeds 20% in those aged 85 years and older. Similarly, age was identified as a risk factor for anemia based on the general population in Minnesota.^18^ Moreover, this study confirmed that rates of anemia were 1.34-fold higher in men than women, which is consistent with the results of our study.^18^ The CCI score has been widely utilized by health researchers to measure burden of disease and case mix. Prior studies have shown that the CCI is a reliable risk predictor of mortality for various conditions including cancer, chronic renal failure, stroke, diabetes, and hip fracture.^19–22^ In addition, although not originally designed for the prediction of resource use, the CCI has also been shown to associate with measures of morbidity including disability, readmission, and length of stay.^23,24^ Nonetheless, no prior research has investigated the direct association between the CCI and anemia. Our study demonstrated that the CCI score displayed as an independent risk factor for anemia on admission in hip fracture patients. Similarly, a large cohort study based on The Third National Health and Nutrition Examination Survey (NHANES III) demonstrated the correlation between anemia rates and the number of comorbidities. It showed that anemia rates increased to between 2.5% and 5.5% in persons with 1, 2, or 3 pathological conditions, like hypertension, diabetes, heart failure, cancer, and so on, and are higher than 6% in those with 4 or more conditions.^25^ These results are consistent with our study.

Variables related to inpatient interventions were major risk factors for postoperation anemia. As shown in the univariate analyses, external fixator, intramedullary fixation, and plate fixation increased the risk of postoperation anemia. Prior research also demonstrated an association between surgical procedures and postoperative blood loss. Kumar et al^26^ indicated that intramedullary fixation was associated with the maximum drop in Hb. However, the multivariate analyses in our study showed no significant associations between surgical procedures and postoperation anemia. Because blood loss during the operation was also included in the multivariate analyses, the effect of surgical procedure may be adjusted by this more powerful and direct variable. Additionally, our results showed that surgical duration >110 minutes was predictive of postoperation anemia, as was blood transfusion, blood loss >200 mL during the operation, which has not been discussed by previous studies. By contrast, variables that demonstrated a significant association with anemia on admission were no longer predictive of postoperation anemia, with the exception of intertrochanteric fracture, as discussed below. These results imply that postoperation anemia mainly resulted from acute hemodynamic changes caused by inpatient interventions, while anemia on admission may be more representative of underlying comorbid illness burden and physiological reserve.

Anemia on discharge was associated with a more mixed set of risk factors. Blood transfusion, surgical duration, and drainage volume were no longer associated with anemia at this time point, while male sex was associated with a significant increase of anemia risk compared to female sex, so was blood loss during the operation and intertrochanteric fracture. The reason for these changes may because by the time of discharge, patients were undergoing recovery, influences of inpatient interventions on hemodynamic changes began to fade. Instead, the ability to replenish blood volume, mainly affected by basic health status, may have become the major risk factor.^27^ However, further research is needed to confirm this possibility.

Interestingly, intertrochanteric fracture remained a risk factor for anemia at all 3 time points. Prior epidemiological studies have demonstrated that patients with a trochanteric fracture were generally older and frailer than patients with a cervical fracture,^28^ which may partly explain its association with admission anemia. However, in our study, trochanteric fracture remained a risk factor for anemia on admission even after adjustment for age, sex, and CCI score, which suggests that there may be other reasons behind this phenomenon. Other studies have also confirmed many differences between trochanteric fracture and femoral neck fracture; however, further research is needed.^29,30^ Additionally, research by Foss and Kehlet^31^ suggested that hidden blood loss associated with surgical treatment for intertrochanteric fracture was substantial in the perioperative period. The Hb levels of patients with intertrochanteric fracture who underwent surgery could not be restored to normal levels at the 7th postoperative day.^14^ These findings may explain the relationships between trochanteric fracture and anemia postoperation and on discharge.

Further analyses into the associations between outcomes and anemia at different time points were also performed. A significant increase in the probability of death for the anemia group was only observed when applying anemia on admission for mortality risk prediction. Also, after adjustments for sex, age, CCI score, classification of fracture, surgical procedure, and general anesthesia, anemia on admission remained an independent risk factor for higher mortality. This result is consistent with other studies focusing on preoperative anemia (or Hb) and hip fracture outcomes.^13,32–34^ Additionally, some other investigators also failed to find an association between postoperative anemia and mortality, while others suggested that postoperation anemia might be a risk factor for complications, readmission, and length of hospital stay.^4,12,35,36^

To sum up, because risk factors for inpatient anemia varies with the time point of diagnosis, when applying anemia in risk prediction of hip fracture mortality, the certain time point should be chosen. Our study suggested that anemia on admission was an independent risk factor for hip fracture mortality, while postoperation and discharge anemia showed no association with mortality risk. The results implicated that:

1. In clinical practice, only admission anemia could be applied for identifying patients at high risk for death while the other 2 time points should not be used.
2. Risk factors for anemia varied at different time points. A patient with any of following characteristics including age >80 years, male sex, high CCI score, and intertrochanteric fracture should be considered at high risk of developing admission anemia; therefore, adequate measures like erythropoietin injection or blood transfusion may be used to prevent anemia.
3. Inpatient interventions contribute greatly to postoperation and discharge anemia; therefore, corresponding measures should be taken to reduce blood loss caused by surgical process. Nonetheless, both postoperation and discharge anemia showed no association with mortality risk.

We acknowledge some limitations to our study. First, as a single-center study, admission bias may present in our sample due to the inherent defect of this type of study. Thus, our findings should be confirmed in other datasets, ideally prospective multicenter studies. Second, a more rigorous methodology to determine the Hb levels of each patient should be to collect every blood sample and assess them uniformly. By contrast, our study could not collect data in this manner, because data for this study were derived from a large database in which collection of blood samples was not a requirement according to the initial design of the database. Nonetheless, standard procedures were performed for complete blood cell counts testing in our hospital, and calibration records indicate the consistency of our results. Moreover, the anemia rates in the present study are comparable with those of other investigations focusing on this issue. Thus, the influence of this limitation may be small. Third, the cutoff values for some variables, such as surgical duration over/under 110 minutes, in our study were the median values. Although significant differences were observed, further investigations are necessary to determine the best cutoff value for risk prediction.

## CONCLUSION

Our study showed that risk factors for anemia varied at different time points, and therapy interventions would greatly affect the status of postoperation and discharge anemia. The take-home message is when anemia is used for mortality prediction in these patients, a specific time point should be chosen. We suggest that only admission anemia should be used for mortality prediction, but not postoperation nor discharge anemia.
